# Supplementary material for: Novel polymycoviruses are encapsidated in filamentous virions
Source: J Virol. 2024 Dec 10;99(1):e01515-24. doi: 10.1128/jvi.01515-24 (PMC11784019; doi:10.1128/jvi.01515-24)
Supplement: Supplemental figures — Fig. S1 to S7. [file jvi.01515-24-s0001.docx]

**
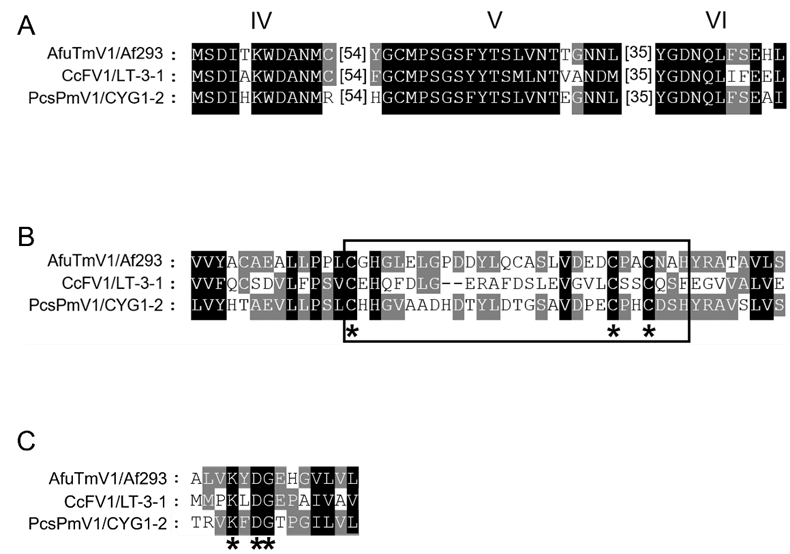
**

**Fig. S1** **Amino acid sequence alignment of PcsPV1 ORFs.** (A) Alignment of the amino acid sequences in the RdRp regions of PcsPV1 ORF1 and selected RNA viruses. Three conserved RdRP motifs corresponding to motifs IV, V, and VI, respectively, are highlighted with black or grey shading. Black and grey shading indicate identical amino acid residues and those belonging to the same group of consensus sequences, respectively. Numbers within brackets indicate the number of amino acids not displayed. (B) Alignment of the amino acid sequence in zinc finger-like motifs of regions on proteins of unknown function putatively encoded by PcsPmV1 ORF2. The motifs are framed, and asterisks signify identical amino acid residues. (C) Alignment of the amino acid sequences in regions on putative methyl transferase (Met) proteins encoded by PcsPmV1 ORF3. Asterisks denote identical amino acid residues.


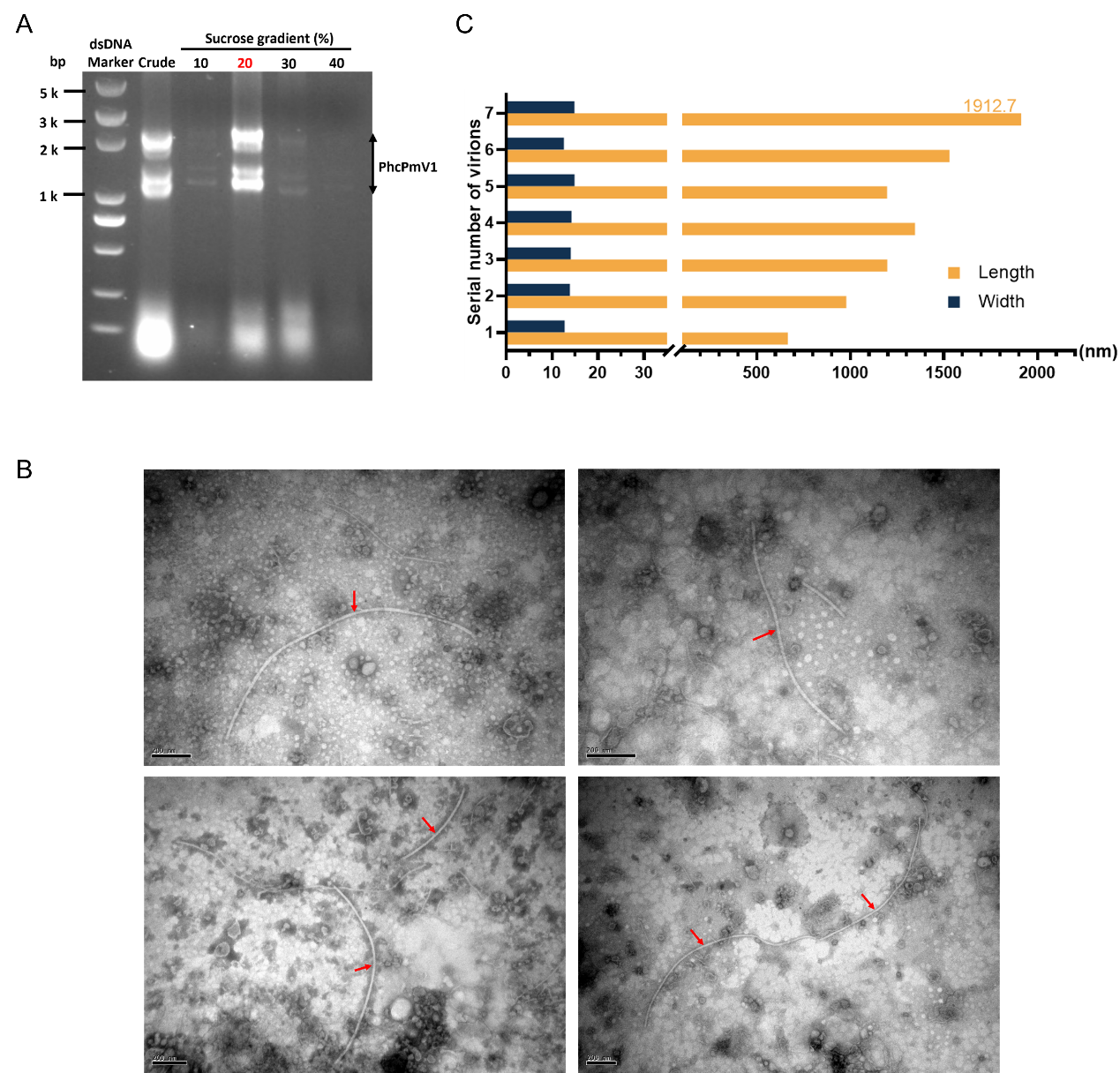


**Fig. S2 Virus-like particles associated with PhcPmV1 from strain DHP2-1.** (A) Agarose gel electrophoresis analysis of dsRNAs extracted from purified virus-like particles from 10% to 40% sucrose fractions at 10% increments from strain DHP2-1 (lane 3-6). (B) Representative virus-like particles extracted from strain DHP2-1 corresponding to the 20% fraction following sucrose gradient centrifugation. (C) Histogram showing the sizes of virus-like particles longer than 500.0 nm in fractions corresponding to 20% sucrose following sucrose gradient centrifugation. The numbers on the vertical axis represent counts of virus-like particles.


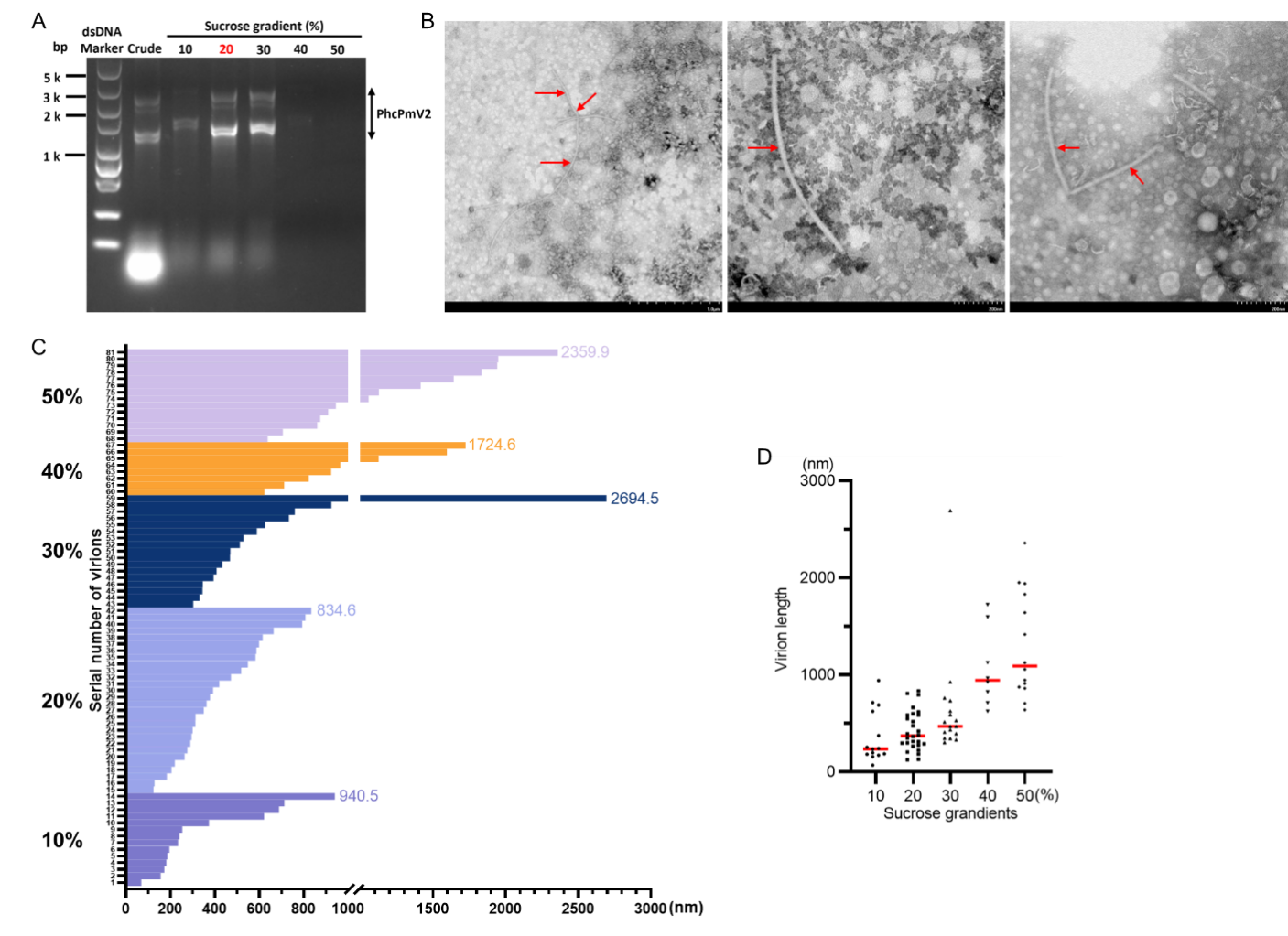


**Fig. S3 Virus-like particles associated with PhcPmV2 from strain FD9-1.** (A) Agarose gel electrophoresis analysis of dsRNAs extracted from purified virus-like particles from 10% to 50% sucrose fractions at 10% increments from strain FD9-1 (lane 3-7). (B) Representative virus-like particles extracted from strain FD9-1 corresponding to the 20% fraction following sucrose gradient centrifugation. (C) Histogram showing the sizes of virus-like particles following sucrose gradient centrifugation. The numbers on the vertical axis represent counts of virus-like particles. (D) Scatter diagram for particle sizes corresponding to 10%—50% sucrose fractions and red lines indicate medians of particle sizes for each sucrose fractions.


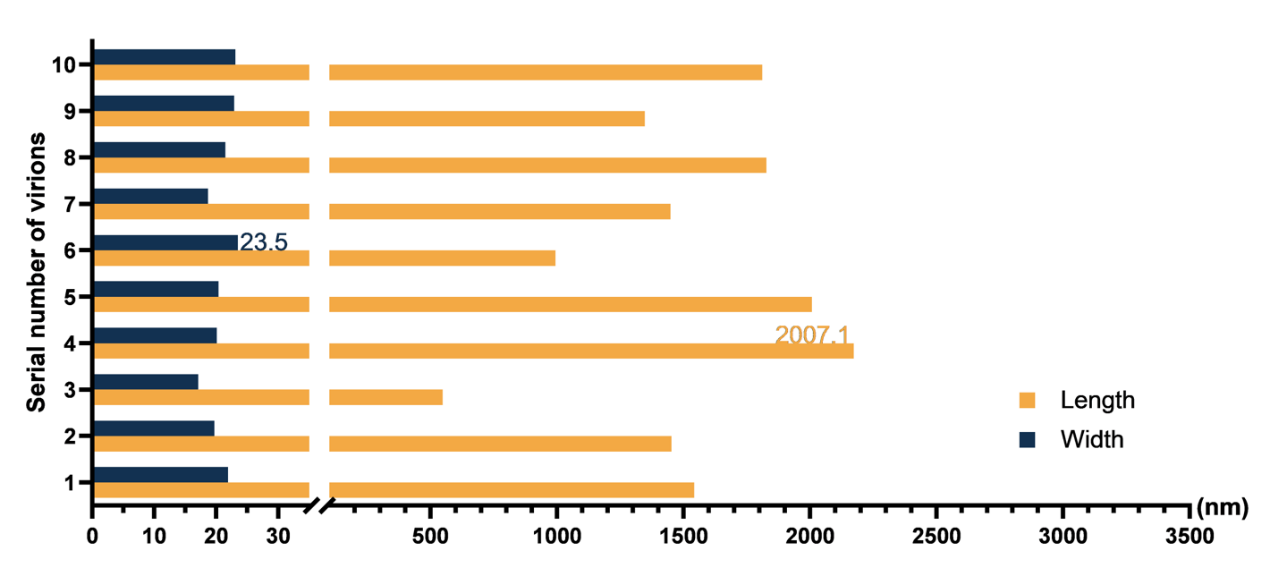


**Fig. S4 Histogram showing the sizes of decorated particles longer than 500.0 nm in fractions corresponding to 30% and 40% sucrose following sucrose gradient centrifugation.** The numbers on the vertical axis represent counts of virus-like particles.


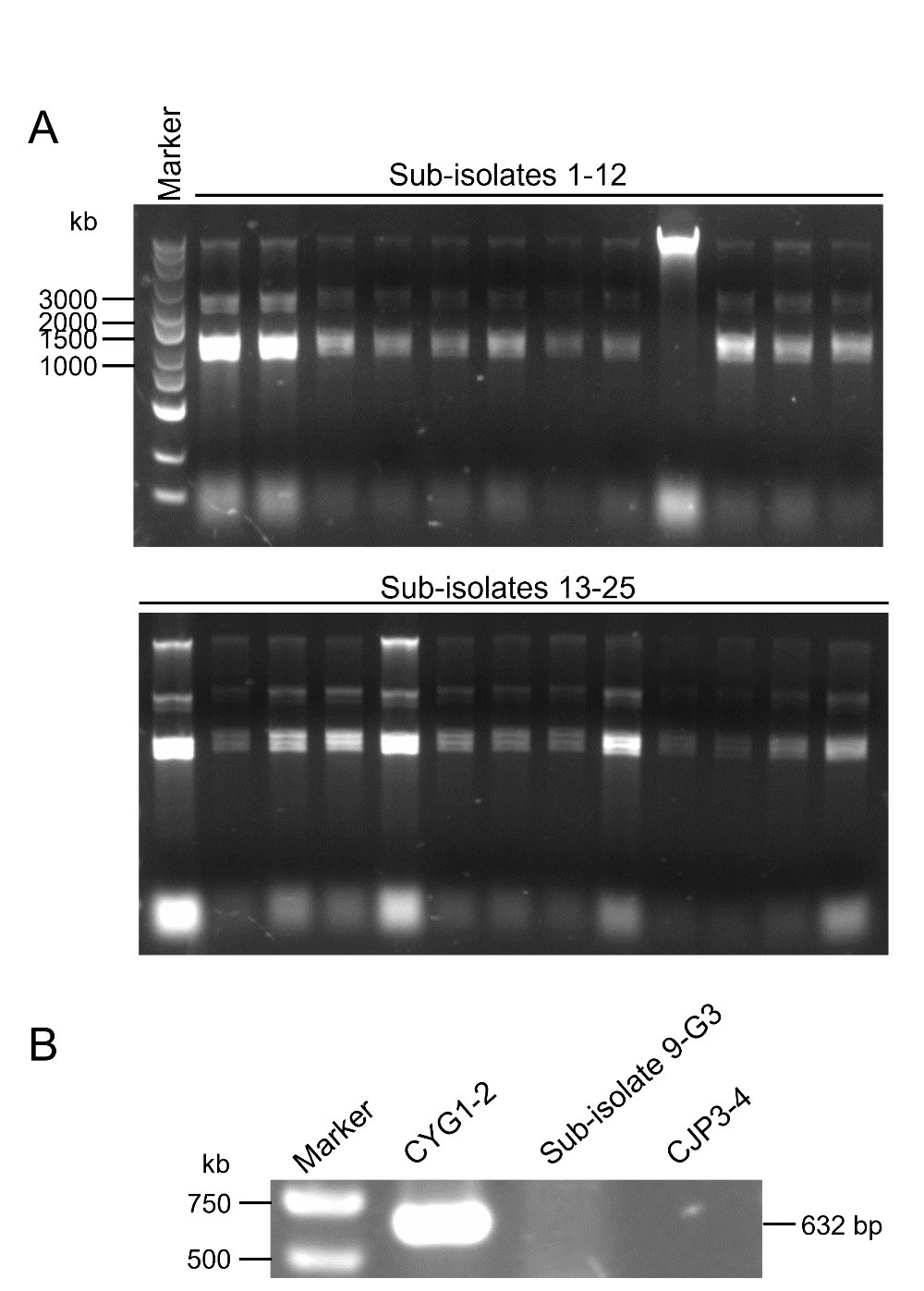


**Fig. S5 Electrophoresis analysis of vertical transmission of PcsPmV1.** (A) Electrophoresis analysis of dsRNAs extracted from the conidium-generated subisolates of strain CYG1-2 on 1.5% agarose gel after treatment with S1 nuclease. (B) RT-PCR detection using primer pair PcsPV1-1-1130F/1761R to detect PfcsPmV1 in the third generation of subisolate 9.


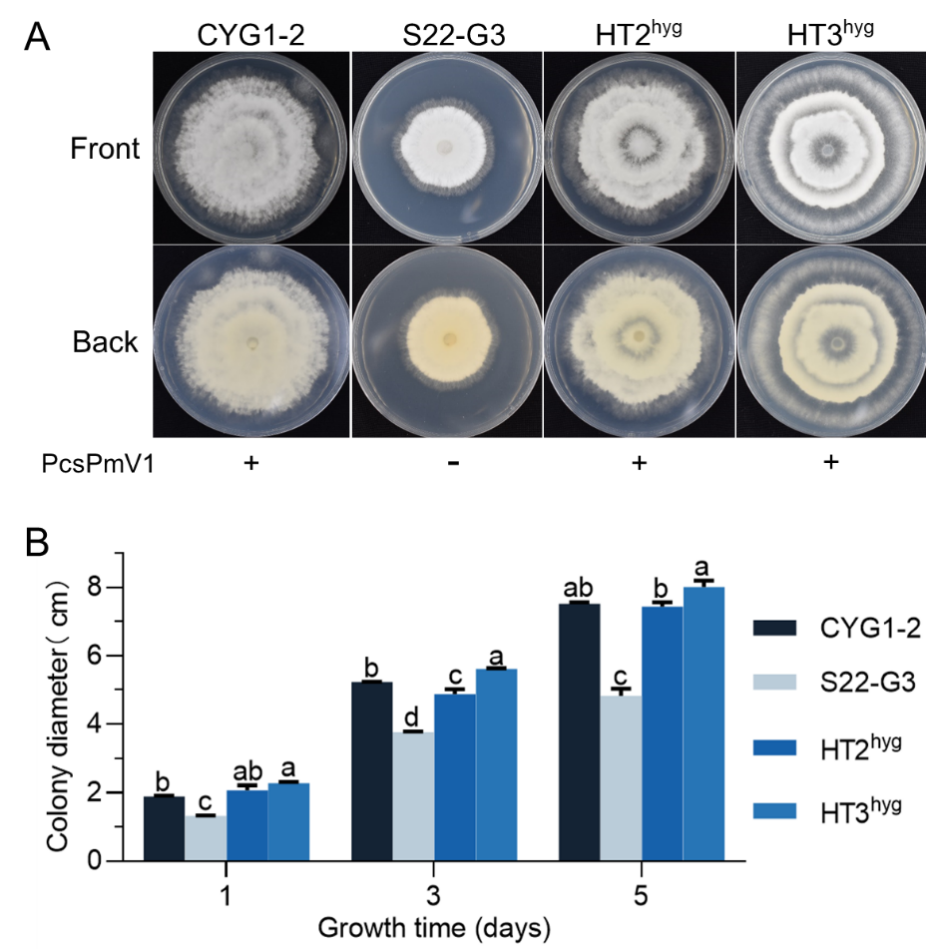


**Fig. S6 Measurement of colony morphology and growth rate of different fungal hosts.** (A and B) Morphologies (A) and colony diameter (B) of *Ps. camelliae-sinensis* strain CYG1-2 and its PcsPmV1-free subisolate (S22-G3) grown on PDA medium for 5 days, respectively. (C) Morphologies of CYG1-2 and CYG1-2-S9 after 5 days of growth on PDA media supplemented with various cellular stress agents. Different letters indicate a significant difference at *p* < 0.05 (one way analysis of variance，One-way ANOVA).

**
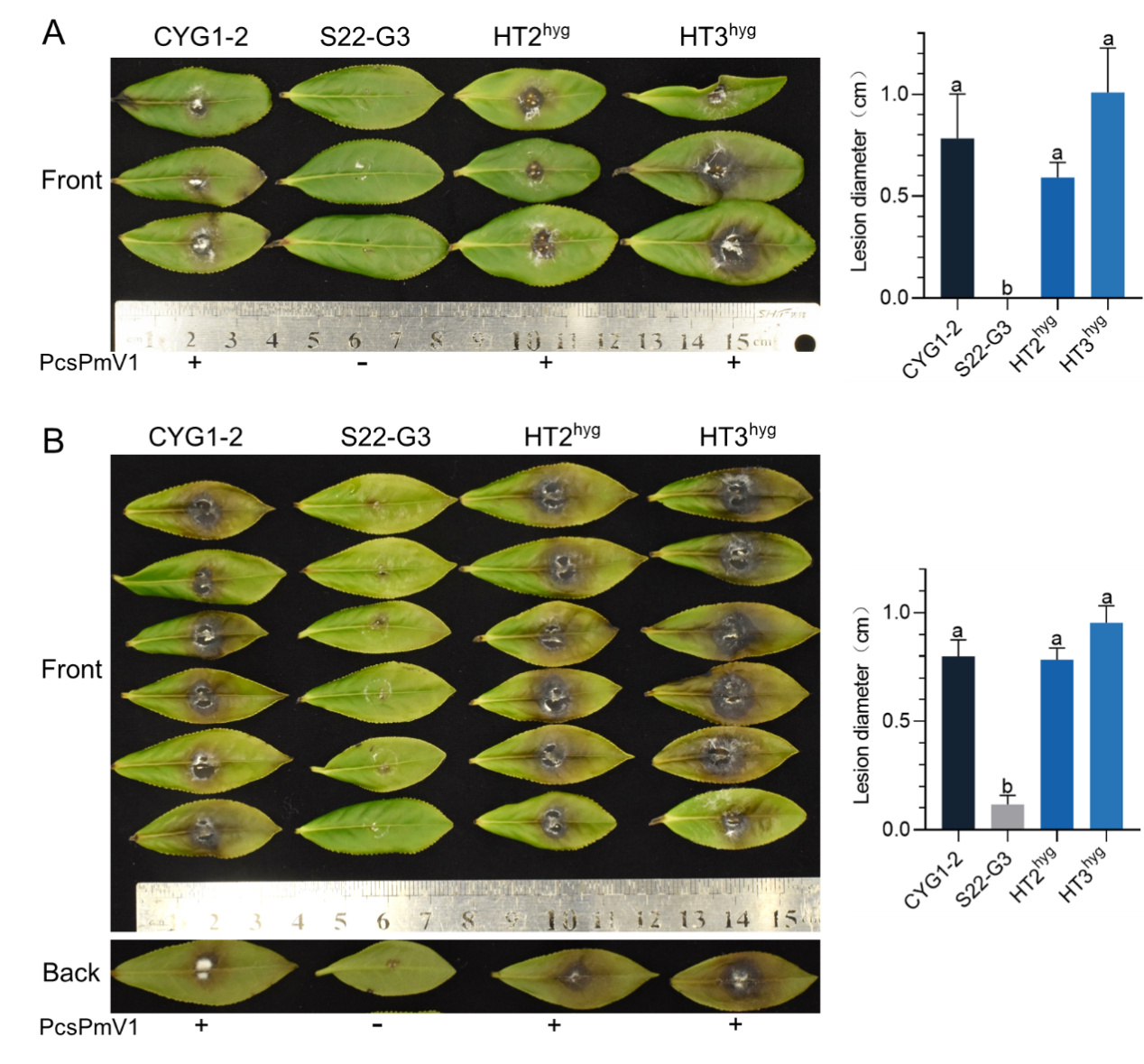
**

**Fig. S7 Measurement of** **pathogenicity of different fungal strains.** (A) Lesions (A) and lesion diameters of PcsPmV1-infected strain CYG1-2, PcsPmV1-free strain S22-G3, PcsPmV1-reinfected strain HT2^hyg^ and HT3^hyg^ infected tea leaves (*C. sinensis* var. E’cha no.1) for 4 days, respectively. (B) Lesions and lesion diameters of four fungal strains infected tea leaves (*C. sinensis* var. Huangyan) for 3 days, respectively. Different letters indicate a significant difference at *p* < 0.05 (One-way ANOVA).
