# Supplementary material for: Novel polymycoviruses are encapsidated in filamentous virions
Source: J Virol. 2024 Dec 10;99(1):e01515-24. doi: 10.1128/jvi.01515-24 (PMC11784019; doi:10.1128/jvi.01515-24)
Supplement: Supplemental tables — Tables S1 to S5. [file jvi.01515-24-s0002.docx]

**Table S1. A list of primers used in this study.**

| **Primer Name** | **Oligonucleotide sequence (5′- 3′)** | **Usage** |
| --- | --- | --- |
| PC3-T7loop | GGATCCCGGGAATTCGGTAATACGACTCACTATATTTTTATAGTGAGTCGTATTA | For RLM-RACE of PcsPmV1 |
| PC2 | CCGAATTCCCGGGATCC |  |
| 05RACE-3RT | CGATCGATCATGATGCAATGCNNNNNN | For random cloning of PcsPmV1 |
| 05RACE-3 | CGATCGATCATGATGCAATGC |  |
| PcsPV1 RdRp-5RACE | TGTTAACACGGAAGGCGCTG | For terminal cloning of PcsPmV1 dsRNA1 |
| PcsPV1 RdRp-3RACE | ATTTCATCTGCGGCCAAGTC |  |
| PcsPV1 dsR2-5RACE | ACCGACGAATATCAGCATAG | For terminal cloning of PcsPmV1 dsRNA2 |
| PcsPV1 dsR2-3RACE | CGTTCTCGCTTTATTTGTCG |  |
| PcsPV1 dsR3-5RACE | TGAGGGCGAGTGTGTTGATG | For terminal cloning of PcsPmV1 dsRNA3 |
| PcsPV1 Met-3RACE | TTACCTTTGTTGACACCTCC |  |
| PcsPV1 PAS1-5RACE | TTGATGACCGCGCCAAGCTC | For terminal cloning of PcsPmV1 dsRNA4 |
| PcsPV1 PAS1-3RACE 2nd | AAGCGACATTCCAGACCGG |  |
| PcsPV1 dsR5-5RACE | ACAAATGACCAGGCCTGAAT | For terminal cloning of PcsPmV1 dsRNA5 |
| PcsPV1 dsR5-3RACE | GACATCCCTGAGAAAGTCTC |  |
| PcsPV1 dsR6 primer1 | GTGAGTAGTGCACTGGTTCG | For terminal cloning of PcsPmV1 dsRNA6 |
| PcsPV1 dsR6 primer2 | ATCGTGTCCTGAATCGCTGT |  |
| PcsPV1-1-1130F | CGACATCTCCCACTTCCTCC | For detecting of PcsPmV1 dsRNA1 |
| PcsPV1-1-1761R | CAGTCTCCTTCACCTTCAGC |  |

**Table S2 Summary of BLASTp searches ORF-coding proteins of PcsPmV1** **with those deposited in NCBI.**

| **ORF serial number** | **Proteins** | **Organism Name** | **Max Score** | **Total Score** | **Query Cover** | **E value** | **Per. Ident** | **Acc. Len** | **Accession** |
| --- | --- | --- | --- | --- | --- | --- | --- | --- | --- |
| 1 | RNA-dependent RNA polymerase [Metarhizium brunneum polymycovirus 1] | Metarhizium brunneum polymycovirus 1 | 869 | 869 | 99% | 0.0 | 57.01% | 767 | WBL45227.1 |
| 1 | RNA-dependent RNA polymerase [Phaeoacremonium minimum tetramycovirus 1] | Phaeoacremonium minimum tetramycovirus 1 | 867 | 867 | 99% | 0.0 | 58.93% | 764 | QDB74985.1 |
| 1 | RdRp [Plasmopara viticola lesion associated polymycovirus 1] | Plasmopara viticola lesion associated polymycovirus 1 | 786 | 786 | 94% | 0.0 | 55.80% | 763 | QHG11067.1 |
| 1 | RNA-dependent RNA polymerase [Alternaria alternata polymycovirus 1] | Alternaria alternata polymycovirus 1 | 785 | 785 | 94% | 0.0 | 55.80% | 763 | QVK45096.1 |
| 1 | ORF1a [Magnaporthe oryzae polymycovirus 1] | Magnaporthe oryzae polymycovirus 1 | 781 | 781 | 100% | 0.0 | 53.25% | 766 | YP_010086046.1 |
| 1 | RNA-dependent RNA polymerase [Magnaporthe oryzae polymycovirus 1] | Magnaporthe oryzae polymycovirus 1 | 778 | 778 | 100% | 0.0 | 52.99% | 766 | QVU39977.1 |
| 1 | RNA-dependent RNA polymerase [Aspergillus fumigatus polymycovirus 1] | Aspergillus fumigatus polymycovirus 1 | 755 | 755 | 100% | 0.0 | 52.34% | 763 | YP_009551547.1 |
| 1 | RNA dependent RNA polymerase [Aspergillus fumigatus polymycovirus 1] | Aspergillus fumigatus polymycovirus 1 | 746 | 746 | 100% | 0.0 | 51.82% | 763 | BBU42080.1 |
| 1 | RNA-dependent RNA polymerase [Talaromyces amestolkiae polymycovirus 1] | Talaromyces amestolkiae polymycovirus 1 | 745 | 745 | 88% | 0.0 | 57.92% | 674 | UYK54776.1 |
| 1 | RNA dependent RNA polymerase [Aspergillus fumigatus tetramycovirus 1] | Aspergillus fumigatus tetramycovirus 1 | 744 | 744 | 100% | 0.0 | 51.82% | 763 | YP_010839797.1 |
| 2 | hypothetical protein [Metarhizium brunneum polymycovirus 1] | Metarhizium brunneum polymycovirus 1 | 676 | 676 | 99% | 0.0 | 51.22% | 699 | WBL45228.1 |
| 2 | hypothetical protein [Alternaria sp. FA0703] | Alternaria sp. FA0703 | 606 | 606 | 99% | 0.0 | 46.77% | 696 | ACL80752.1 |
| 2 | hypothetical protein [Alternaria alternata polymycovirus 1] | Alternaria alternata polymycovirus 1 | 606 | 606 | 99% | 0.0 | 46.77% | 696 | QVK45097.1 |
| 2 | putative serine protease [Phaeoacremonium minimum tetramycovirus 1] | Phaeoacremonium minimum tetramycovirus 1 | 597 | 597 | 100% | 0.0 | 46.77% | 696 | QDB74986.1 |
| 2 | hypothetical protein [Aspergillus fumigatus polymycovirus 1] | Aspergillus fumigatus polymycovirus 1 | 585 | 585 | 99% | 0.0 | 46.79% | 696 | BCH36614.1 |
| 2 | hypothetical protein QK611_s2gp1 [Aspergillus fumigatus tetramycovirus 1] | Aspergillus fumigatus tetramycovirus 1 | 582 | 582 | 99% | 0.0 | 46.09% | 696 | YP_010839794.1 |
| 2 | protease [Aspergillus fumigatus polymycovirus 1] | Aspergillus fumigatus polymycovirus 1 | 582 | 582 | 99% | 0.0 | 46.23% | 696 | YP_009551545.1 |
| 2 | hypothetical protein [Aspergillus fumigatus polymycovirus 1] | Aspergillus fumigatus polymycovirus 1 | 581 | 581 | 99% | 0.0 | 46.23% | 696 | BBU42081.1 |
| 2 | hypothetical protein [Talaromyces amestolkiae polymycovirus 1] | Talaromyces amestolkiae polymycovirus 1 | 560 | 560 | 99% | 0.0 | 44.83% | 695 | UYK54777.1 |
| 2 | P2 [Setosphaeria turcica polymycovirus 1] | Setosphaeria turcica polymycovirus 1 | 554 | 554 | 99% | 0.0 | 43.84% | 697 | UMZ55611.1 |
| 3 | methyl transferase [Metarhizium brunneum polymycovirus 1] | Metarhizium brunneum polymycovirus 1 | 554 | 554 | 100% | 0.0 | 47.97% | 615 | WBL45229.1 |
| 3 | methyl transferase [Talaromyces amestolkiae polymycovirus 1] | Talaromyces amestolkiae polymycovirus 1 | 489 | 489 | 100% | 2e-162 | 46.02% | 613 | UYK54778.1 |
| 3 | Methyl transferase [Aspergillus fumigatus polymycovirus 1] | Aspergillus fumigatus polymycovirus 1 | 474 | 474 | 100% | 2e-156 | 43.71% | 622 | BBU42082.1 |
| 3 | Methyl transferase [Aspergillus fumigatus tetramycovirus 1] | Aspergillus fumigatus tetramycovirus 1 | 472 | 472 | 100% | 9e-156 | 43.55% | 614 | YP_010839796.1 |
| 3 | hypothetical protein [Aspergillus fumigatus polymycovirus 1] | Aspergillus fumigatus polymycovirus 1 | 470 | 470 | 100% | 6e-155 | 43.30% | 622 | BCH36615.1 |
| 3 | methyl transferase [Aspergillus fumigatus polymycovirus 1] | Aspergillus fumigatus polymycovirus 1 | 469 | 469 | 100% | 8e-155 | 43.18% | 622 | YP_009551546.1 |
| 3 | putative methyltransferase [Phaeoacremonium minimum tetramycovirus 1] | Phaeoacremonium minimum tetramycovirus 1 | 426 | 426 | 81% | 1e-139 | 49.90% | 501 | QDB74987.1 |
| 3 | hypothetical protein [Magnaporthe oryzae polymycovirus 1] | Magnaporthe oryzae polymycovirus 1 | 412 | 412 | 99% | 2e-132 | 42.14% | 612 | QVU39979.1 |
| 3 | methyltransferase [Alternaria alternata polymycovirus 1] | Alternaria alternata polymycovirus 1 | 411 | 411 | 90% | 4e-132 | 42.53% | 614 | QVK45098.1 |
| 3 | methyl transferase [Plasmopara viticola lesion associated polymycovirus 1] | Plasmopara viticola lesion associated polymycovirus 1 | 406 | 406 | 90% | 2e-130 | 42.10% | 614 | QHG11068.1 |
| 5 | putative PAS-rich protein [Phaeoacremonium minimum tetramycovirus 1] | Phaeoacremonium minimum tetramycovirus 1 | 277 | 277 | 95% | 2e-89 | 53.82% | 272 | QDB74988.1 |
| 5 | proline-alanine-serine-rich protein |  | 250 | 250 | 96% | 5e-79 | 47.15% | 265 | UXC94315.1 |
| 5 | hypothetical protein [Beauveria bassiana polymycovirus 4] | Beauveria bassiana polymycovirus 4 | 249 | 249 | 96% | 9e-79 | 46.77% | 265 | QRF54816.1 |
| 5 | proline-alanine-serine rich protein [Beauveria bassiana polymycovirus 3] | Beauveria bassiana polymycovirus 3 | 248 | 248 | 96% | 5e-78 | 46.99% | 268 | CAD7829826.1 |
| 5 | hypothetical protein [Alternaria alternata polymycovirus 1] | Alternaria alternata polymycovirus 1 | 244 | 244 | 98% | 1e-76 | 46.67% | 269 | QVK45100.1 |
| 5 | hypothetical protein [Erysiphe necator associated polymycovirus 6] | Erysiphe necator associated polymycovirus 6 | 244 | 244 | 96% | 2e-76 | 46.59% | 269 | QKK35423.1 |
| 5 | putative PAS-rich protein [Erysiphe necator associated polymycovirus 4] | Erysiphe necator associated polymycovirus 4 | 238 | 238 | 95% | 2e-74 | 47.35% | 266 | QKK35416.1 |
| 5 | PAS rich protein [Talaromyces amestolkiae polymycovirus 1] | Talaromyces amestolkiae polymycovirus 1 | 227 | 227 | 95% | 5e-70 | 44.91% | 264 | UYK54780.1 |
| 5 | P-A-S-rich protein [Botryoshaeria dothidea polymycovirus 1] | Botryoshaeria dothidea polymycovirus 1 | 227 | 227 | 93% | 1e-69 | 49.61% | 275 | UVZ34696.1 |
| 5 | hypothetical protein [Diplodia seriata polymycovirus 1] | Diplodia seriata polymycovirus 1 | 221 | 221 | 95% | 2e-67 | 47.57% | 275 | UOK20168.1 |

**Table S3 Summary of the peptide mass fingerprinting analysis of P28 encoded by ORF5 of PcsPmV1.**

| **Amino Acid Position** | **Calculated Mass** | **Observed Mass** | **ppm** | **Amino Acid Sequence** | **Ion Score** |
| --- | --- | --- | --- | --- | --- |
| 2-14 | 1490.7439 | 746.3829 | 5 | M.AFSNILSPELCAK.L | 89 |
| 15-28 | 1399.7922 | 700.9067 | 5 | K. LSAITADELGAVIK.A | 91 |
| 15-38 | 2354.3169 | 1178.1697 | 3 | K. LSAITADELGAVIKAASLGLSAQR.L | 64 |
| 29-38 | 972.5352 | 487.2750 | 0 | K. AASLGLSAQR.L | 65 |
| 39-73 | 3814.8989 | 1272.6307 | -7 | R. LHECIHAVNSGESPELPEVSGSPKPVTVQAWSFVK.D | 50 |
| 39-75 | 4086.0269 | 1363.0120 | -3 | R. LHECIHAVNSGESPELPEVSGSPKPVTVQAWSFVKDR.G | 1 |
| 74-102 | 3169.5415 | 1057.5216 | 0 | K. DRGQYADTYQLSSAQAGEIADLLLTDVEK.G | 85 |
| 76-102 | 2899.3974 | 967.4824 | 0 | R. GQYADTYQLSSAQAGEIADLLLTDVEK.G | 62 |
| 103-116 | 1413.8191 | 703.9193 | 4 | K. GIAEITAIVTSALR.R | 91 |
| 103-117 | 1569.9202 | 785.9716 | 5 | K. GIAEITAIVTSALRR.R | 41 |
| 118-135 | 1799.9424 | 450.9930 | 0 | R. RGSARPVAVSTAGMPATR.G | 42 |
| 119-135 | 1643.8413 | 548.9551 | 1 | R. GSARPVAVSTAGMPATR.G | 76 |
| 136-149 | 1244.6626 | 623.3394 | 1 | R. GAPPPVPGVGNAGR.G | 60 |
| 150-157 | 914.5185 | 458.2663 | -0 | R. GALKEEIR.S | 55 |
| 158-167 | 1086.5345 | 544.2752 | 1 | R. SNAATYGLYK.F | 49 |
| 158-180 | 2488.1982 | 830.4105 | 5 | R. SNAATYGLYKFDAEDTGRPGSLR.Y | 62 |
| 168-180 | 1419.6743 | 710.8447 | 0 | K. FDAEDTGRPGSLR.Y | 30 |
| 185-196 | 1199.6299 | 600.8229 | 1 | R. LGGGLYATHPSK.T | 70 |
| 185-204 | 1983.0538 | 662.0270 | 3 | R. LGGGLYATHPSKTGAIDVAR.I | 60 |
| 197-204 | 801.4345 | 401.7250 | 1 | K. TGAIDVAR.I | 55 |
| 212-219 | 917.4970 | 459.7569 | 2 | R. AYPLIADR.I | 30 |
| 220-227 | 1006.5488 | 504.2829 | 2 | R. IYLWIDGK.T | 39 |
| 220-238 | 2115.1001 | 1058.5574 | 0 | R. IYLWIDGKTPAVGSDIPDR.V | 102 |
| 228-238 | 1126.5619 | 564.2900 | 3 | K. TPAVGSDIPDR.V | 81 |
| 228-258 | 3202.6874 | 801.6850 | 7 | K. TPAVGSDIPDRVTFTGILPPLADLPADPTTR.A | 45 |
| 239-258 | 2094.1361 | 1048.0794 | 4 | R. VTFTGILPPLADLPADPTTR.A | 62 |

**Table S4 Summary of the peptide mass fingerprinting analysis with ion score more than 18 of p82.**

| Species | Protein | Amino acid position | Calculated Mass | Observed Mass | ± delta | Amino acid sequence | Ion score | GenBank accession no. |
| --- | --- | --- | --- | --- | --- | --- | --- | --- |
| *Macrophomina phaseolina* | Glycoside hydrolase family 15 | 139–155 | 1991.9966 | 1993.1091 | 0.1052 | FNVDLTQFTGAWGRPQR | 40 | gi\|407919636 |
|  |  | 162–182 | 2355.0953 | 2356.2378 | 0.1352 | ATSMIAYAHWLINNGYSDTAR | 18 | gi\|407919637 |
|  |  | 162–182 | 2371.0903 | 2372.2332 | 0.1356 | ATSMIAYAHWLINNGYSDTAR | 44 | gi\|407919638 |
|  |  | 183–190 | 1014.5498 | 1015.6092 | 0.0521 | DVVWPVIR | 35 | gi\|407919639 |
|  |  | 606–620 | 1683.7489 | 1684.8444 | 0.0882 | KSSSGAYTWESDPNR | 41 | gi\|407919640 |
|  |  | 607–620 | 1555.6539 | 1556.7433 | 0.0821 | SSSGAYTWESDPNR | 61 | gi\|407919641 |
| *Botrytis cinerea* | hypothetical protein | 72–82 | 1463.6357 | 1464.7218 | 0.0788 | TNPDYFYTWTR | 81 | gi\|154303741 |
|  |  | 72–82 | 1495.6256 | 1496.7101 | 0.0772 | TNPDYFYTWTR | 33 | gi\|154303742 |
| *Sporothrix schenckii* | hypothetical protein | 65–75 | 1463.6357 | 1464.7218 | 0.0788 | TNPNYFYTWTR | 81 | gi\|550805530 |
|  |  | 65–75 | 1495.6255 | 1496.7101 | 0.0773 | TNPNYFYTWTR | 33 | gi\|550805531 |
| *Aureobasidium pullulans* | glucoamylase protein | 72-82 | 1463.6357 | 1464.7218 | 0.0788 | TDPNYFYTWTR | 81 | gi\|662524132 |
|  |  | 72-82 | 1495.6256 | 1496.7101 | 0.0772 | TDPNYFYTWTR | 33 | gi\|662524133 |
| *Pseudogymnoascus pannorum* | hypothetical protein | 75-85 | 1463.6358 | 1464.7218 | 0.0787 | TDPDYFYTWTR | 81 | gi\|682270170 |
|  |  | 75-85 | 1495.6256 | 1496.7101 | 0.0772 | TDPDYFYTWTR | 33 | gi\|682270171 |
| *Pseudogymnoascus pannorum* | hypothetical protein | 386-402 | 1884.8489 | 1885.999 | 0.1428 | AQQYTPSNGALAEQYSR | 44 | gi\|682405654 |
| *Cladophialophora yegresii* | hypothetical protein | 411-425 | 1626.859 | 1627.8695 | 0.0032 | APPPINTAHPREATR | 41 | gi\|628299414 |
| *Trichoderma atroviride* | hypothetical protein | 65-72 | 1014.5498 | 1015.6092 | 0.0521 | NVVWPVIR | 37 | gi\|358397680 |
| *Stereum hirsutum* | hypothetical protein | 203-215 | 1610.7318 | 1611.8768 | 0.1377 | MGRDVDLASSSTNSR | 32 | gi\|618814056 |
| *Serendipita vermifera* | carbohydrate-binding module family 20 protein | 563-576 | 1592.7067 | 1593.8541 | 0.1401 | VNGSTVTWESDPNR | 28 | gi\|751675420 |
| *Neosartorya udagawae* | hypothetical protein | 355-366 | 1409.7011 | 1410.7386 | 0.0302 | NLDSDPNPRNLR | 21 | gi\|849267316 |
| *Phaeosphaeria nodorum* | hypothetical protein | 57-176 | 2023.9521 | 2025.101 | 0.1416 | MGLSSLDVDTTTGEAFAPGR | 21 | gi\|169596052 |
| *Saccharomycetaceae sp.* | AaceriAGL063Cp | 261-270 | 1212.6503 | 1213.7302 | 0.0726 | SVYDYIAVKR | 21 | gi\|513033843 |
| *Histoplasma capsulatum* | hypothetical protein | 1093-1115 | 2584.3483 | 2585.4238 | 0.0682 | NDLVDVLNSIKEVQELVIEVASR | 20 | gi\|154284430 |
| *Cryptococcus neoformans* var. grubii H99 | DNA topoisomerase 2-associated protein | 799-815 | 1904.0414 | 1905.1233 | 0.0746 | ILHTVQSARNPMVSPVR | 19 | gi\|799315522 |
| *Baudoinia compniacensis* | hypothetical protein | 843-866 | 2993.2854 | 2994.4771 | 0.1844 | QHQDCNMQIVNCTTPSNSFHIFRR | 18 | gi\|627802221 |

**Table S5 Summary of the peptide mass fingerprinting analysis of p28 encoded by ORF4 of BdRV1.**

| Amino acid position | Calculated Mass | Observed Mass | ± delta | Amino acid sequence | Ion score |
| --- | --- | --- | --- | --- | --- |
| 2–16 | 1572.8359 | 1573.8235 | -0.0197 | ASTTTTNNGPLLLTK | 41 |
| 17–35 | 1882.0411 | 1883.0336 | -0.0148 | EQASLLAALGEGEVAAIIK | 106 |
| 36–51 | 1728.9886 | 1729.9811 | -0.0148 | LASLGLRPTGILEYAR | 80 |
| 52–80 | 2990.5753 | 2991.5505 | -0.0321 | AVDGDEVPAPPTPNTAAKPVTIVAWSFLK | 2 |
| 81–95 | 1690.8176 | 1691.8125 | -0.0124 | DRGQYHATYGLSAPR | 97 |
| 83–95 | 1419.6895 | 1420.6859 | -0.0109 | GQYHATYGLSAPR | 108 |
| 96–112 | 1740.9257 | 1741.9210 | -0.0120 | AGELLELLLTDADAAAR | 157 |
| 113–123 | 1236.6826 | 1237.6770 | -0.0129 | EIHTIVADALR | 84 |
| 126–141 | 1672.9009 | 1673.8939 | -0.0143 | GSPRPVHVTLDGVPSR | 54 |
| 143–174 | 3079.4847 | 3080.4592 | -0.0328 | KAGGEGAPTGDGGLGLLSQEIASNPGTYGSYR | 193 |
| 144–174 | 2951.3897 | 2952.3613 | -0.0357 | AGGEGAPTGDGGLGLLSQEIASNPGTYGSYR | 246 |
| 175–186 | 1285.6415 | 1286.6406 | -0.0082 | FVPEQTGQPGAR | 69 |
| 225–237 | 1385.6616 | 1386.6512 | -0.0177 | DFVAFWATGSSAK | 109 |
| 238–251 | 1536.7573 | 1537.7527 | -0.0119 | FGGEIPDKVTFDGR | 119 |
| 252-265 | 1510.7263 | 1511.7167 | -0.0169 | QGPNEPLQSDPTTK | 76 |
| Note: The overall protein score is 1541. |  |  |  |  |  |
